# Role of the hedgehog signaling pathway in rheumatic diseases: An overview

**DOI:** 10.3389/fimmu.2022.940455

**Published:** 2022-08-25

**Authors:** Yazhen Su, Hao Xing, Jie Kang, Linkun Bai, Liyun Zhang

**Affiliations:** Third Hospital of Shanxi Medical University, Shanxi Bethune Hospital, Shanxi Academy of Medical Sciences, Tongji Shanxi Hospital, Taiyuan, Shanxi, China

**Keywords:** hedgehog signaling pathway, SMO, GLI, inflammation, target treatment

## Abstract

Hedgehog (Hh) signaling pathway is an evolutionarily conserved signal transduction pathway that plays an important regulatory role during embryonic development, cell proliferation, and differentiation of vertebrates, and it is often inhibited in adult tissues. Recent evidence has shown that Hh signaling also plays a key role in rheumatic diseases, as alterations in their number or function have been identified in rheumatoid arthritis, osteoarthritis, ankylosing spondylitis, systemic sclerosis, and Sjogren’s Syndrome. As a result, emerging studies have focused on the blockade of this pathogenic axis as a promising therapeutic target in several autoimmune disorders; nevertheless, a greater understanding of its contribution still requires further investigation. This review aims to elucidate the most recent studies and literature data on the pathogenetic role of Hh signaling in rheumatic diseases.

## Introduction: An overview of the Hh signaling pathway

The Hedgehog (Hh) gene was first discovered in Drosophila in 1980 by Nusslein-Volhard, C. and Wieschaus, E., and was named Hedgehog because its mutation developed Drosophila larvae into a hedgehog-like morphology, which was also confirmed in vertebrates lately (1). The Hh signaling pathway in vertebrates mainly consists of four parts, including Hh ligands, membrane protein receptors, nuclear transcription factors, and downstream target genes. There are three Hh genes that have been detected: the Sonic Hedgehog (Shh), Desert Hedgehog (Dhh), and Indian Hedgehog (Ihh), which encode the corresponding Shh, Dhh, and Ihh proteins. These three proteins are collectively known as Hh ligands. Among them, Shh is the most widely distributed with a high positive expression rate, which is also the most studied Hh protein in the literature (2); Dhh is an essential regulator in the development of reproductive system (3); Ihh is mainly produced and secreted by prehypertrophic chondrocytes to regulate the growth process of growth plate cartilage (4). Ptched (Ptch) and Smoothened (Smo) are the receptors for Hh protein, existing on the target cell membrane. Ptch is a 12-fold transmembrane protein encoded by the tumor suppressor gene Ptched, which negatively regulates Hh signaling, and vertebrate has two forms of Ptch1 and Ptch2 (5). Smo is one of the family members of seven transmembranes G protein-coupled receptors and is a bioreceptor required for activation of Hh signaling. It is a highly conserved amino acid sequence in transmembrane regions with the N terminus located extracellular, and the C terminus intracellular (5). The nuclear transcription factors are homologous zinc finger structural transcription factors encoded by Gli genes. In vertebrates, there are three members of the Gli gene family: Gli1, Gli2, and Gli3. Gli proteins regulate the expression of target genes by directly binding to their promoters (6). Hh target genes are involved in cell cycle regulation, proliferation, apoptosis, and angiogenesis (7). Furthermore, suppressed fusion protein (Sufu) is a critical intracellular negative regulator of Hh signaling. Sufu inhibits Gli protein by preventing its translocation into the nucleus (8). It plays a crucial role in Gli stabilization and processing.

**Figure 1:**
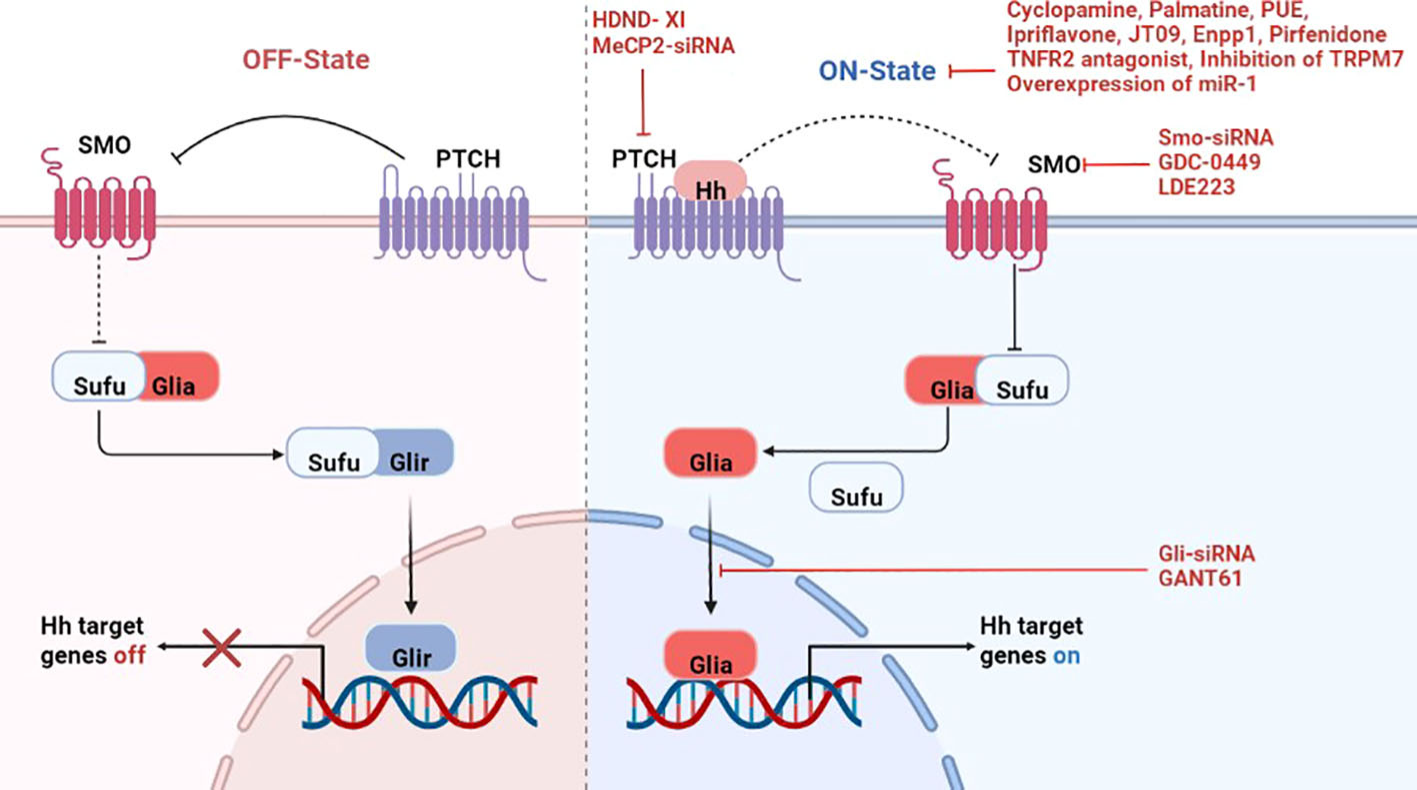
A simplified display of the canonical Hedgehog signaling pathway and treatment of rheumatic diseases by targeting the pathway. Left panel: In the absence of Hh ligands, Ptch suppresses any inactive Smo, leaving Sufu free to bind to Gli activator (Glia), thus repressing it and keeps Hh target genes switch off. Right panel: Binding of Hh ligands to Ptch leading to the dis-inhibition of Smo, which then inhibits Sufu, thereby releasing the nuclear translocation of Glia proteins. Activated Glia in the cytoplasm then translocate into the nucleus and promote transcription of Hh target genes. And there are inhibitors of different genes to suppress the activation of this pathway. Hh-Hedgehog; Ptch-Patched; Smo-Smoothened; Gli-glioma-associated oncogene; Glia-Gli activator; Glir-Gli repressor; Sufu-Suppressed fusion protein; HDND-XI-5-(4-Chlorophenethyl) imino-7-O-acetyl-4-chlorophenethylamine hesperetin; PUE-Phlomis umbrosa extract; Enpp1-Ectonucleotide pyrophosphatase/phosphodiesterase 1.

Activation of Hh signaling includes canonical and non-canonical pathways, the canonical pathway is the most extensively studied and clearly understood currently, which is also known as the ligand-dependent pathway (9). In the absence of Hh ligands, Ptch suppresses Smo, leaving Sufu free to bind to Gli activator (Glia), thus repressing it and keeping Hh target genes switch off. When the Hh ligand binds to Ptch, resulting in loss of inhibition of Smo and then inhibits Sufu, thereby releasing the nuclear translocation of Glia proteins. Activated Glia is transported from the cytoplasm to the nucleus to promote the transcription of Hh target genes (10)(**Figure 1**).

Numerous studies have shown that aberrant expression of Hh signaling may take part in a wide range of diseases, and current studies have confirmed the involvement of this pathway in a variety of tumors. Similarly, scholars have also explored the engagement of the Hh signaling pathway in the pathogenesis (**Table 1**) and targeted therapy (**Table 2**) of rheumatic diseases.

**Table 1:** The expression of Hedgehog signaling in rheumatic diseases.

Disease	Source	Result	Ref
RA	serum	Shh is increased compared with SLE, AS and health controls, it correlates with RF and anti-CCP Ab positively	(11)
	PBMCs	Shh and Gli1 mRNA are increased, and there is no difference in the expression of Ptch1 mRNA compared with health controls	(12)
	synovial tissue	Shh, Smo, and Gli1 protein are higher than health controls	(13)
	FLS	Shh, Ptch, Smo, and Gli are highly expressed	(11)
	chondrocyte	Shh, Ptch1, Smo, and Gli1 proteins are increased in AIA rats cartilage tissue, and the level of these proteins are proportional to the degree of chondrocyte damage	(14)
	endothelial cells	Smo expression is significantly elevated	(15)
OA	chondrocyte	Gli1, Ptch1 expression are increased	(16)
		Ihh is highly expressed	(16–23)
		Shh is expressed	(24)
SSc	skin	Shh, Ptch1, Ptch2, Gli1, Gli2 are increased	(25)
AS	serum	Ihh is higher than RA patients and healthy controls, and its expression is reduced after TNF-antagonist treatment	(26)

**Table 2:** Treatment of rheumatic diseases by targeting the Hh signaling pathway.

Disease	Inhibitor	Target	Result	Ref
RA	Cyclopamine	–	reduces the proliferation and expression of Shh, Smo, Gli1 mRNA in RA-FLSs	(13)
			decreased the expression of Shh, Ptch1, Smo, and Gli1 in AIA rats, while reducing joint inflammation and cartilage damage, decreasing the levels of pro-inflammatory factors in serum, and increasing the levels of COII in articular cartilage.	(14)
			inhibits apoptosis of chondrocytes	(66)
			decreases EA.hy926 endothelial cell viability and survival, and promoted apoptosis.	(15)
	Smo-siRNA	Smo	inhibites the proliferation of endothelial cells and promotes its apoptosis	(15)
	GANT61	Gli	inhibites proliferation in a dose-dependent manner and increases the apoptosis rate of RA-FLSs	(11)
	GANT61	Gli	reduces the level of Shh protein and inhibited proliferation of FLSs in CIA rats	(67, 68)
	GDC-0449	Smo	suppresses the proliferation of FLSs	(69)
	MeCP2-siRNA	Ptch1	inhibites the activation of Hh pathway and decreases the expression of Gli1 and Shh	(70)
	5-(4-Chlorophenethyl) imino-7-O-acetyl-4-chlorophenethylamine hesperetin(HDND- XI)	Ptch1	attenuates AA-FLSs inflammation by reducing the methylation level of the Ptch1 gene	(71)
	TNFR2 antagonist	TL1A	reduces the expression of Ihh and its receptor Ptch1, 2 in RA-FLSs	(72)
	Inhibition of TRPM7	–	attenuate chondrocyte apoptosis and articular cartilage damage by modulating Ihh signaling	(73)
	AMSP-30m	HIF-1α	inhibited SFs proliferation and promoted its apoptosis.it inhibites the activation of Shh pathway	(74)
OA	Ihh knockdown	Ihh	prevents OA progression by inhibiting chondrocyte hypertrophy and collagen type X, MMP-13 and Runx2 expression	(17, 75)
	Smo-siRNA	Smo	reduce the severity of OA	(16)
	Inhibition of Gli1	Gli1	reduces subchondral local immune inflammatory responses and attenuates articular cartilage degeneration in TMJOA mouse model	(76)
	LDE223	Smo	inhibits chondrocyte differentiation, reduces the expression of type X collagen, and inhibits bone hyperplasia in mice	(77)
	Palmatine(Pal)	–	has a protective effect on cartilage and may inhibit MMPs by inhibiting the Hh signaling	(78)
	*Phlomis umbrosa* extract (PUE)	–	improves joint pathology in animal models of OA by modulating the Shh signaling pathway	(79, 80)
	Ipriflavone	–	attenuates the degeneration of cartilage by blocking the Ihh pathway	(81)
	JT09	kappa opioid receptor	reduces cartilage loss and prevented degenerative changes in joints, while reducing the expression of Hh signaling components in cartilage	(82)
	GANT-61 and indomethacin	–	reduces cartilage damage and decreases levels of TNF-α, IL-2, and IL-6 in OA	(83)
	Ectonucleotide pyrophosphatase/phosphodiesterase 1 (Enpp1)	–	inhibits ectopic joint calcification and maintains articular chondrocytes by inhibiting Hh signaling	(84)
	Overexpression of miR-1	–	inhibits the development of OA by inhibiting Ihh signaling	(85)
SSc	Inhibition of Gli	Gli	reduces the expression of CLIC4 in fibroblasts	(86)
	HHAT knockdown	Shh	inhibits TGF-β-induced Hh signaling expression, and suppresses fibroblast activation and tissue fibrosis	(87)
	LDE223	Smo	lighten bleomycin-induced dermal fibrosis and inhibit the aberrant activation of Hh pathway	(88)
	Pirfenidone	–	reduces the Hh signaling expression	(89)

## The role of Hh signaling pathway in rheumatic diseases

### Rheumatoid arthritis

Rheumatoid arthritis (RA) is mainly characterized by chronic inflammation of the synovium and progressive joint destruction. Fibroblast-like synoviocytes (FLSs) are the key effectors that mediate synovitis and joint destruction in RA (27). Abnormal activation and proliferation of FLSs release a large number of cytokines, chemokines, and matrix-degrading enzymes, leading to joint destruction and bone erosion. The relationship between Hh signaling and RA has also been gradually increasing recently.

Compared with systemic lupus erythematosus (SLE), ankylosing spondylitis (AS) and healthy controls, the expression of Shh in serum of RA patients was significantly increased; the level of Shh in RA patients was correlated with rheumatoid factor (RF) and anti-cyclic citrullinated peptide antibodies (anti-CCP Ab) positively, but it had no correlation with erythrocyte sedimentation rate (ESR) (11). Similarly, Wang et al. detected the gene expression in PBMCs of 35 RA patients and found that the relative expression of Shh and Gli1 mRNA were higher than the control group, while there was no difference in the expression of Ptch1 mRNA (12). In addition, academics found the expression of Shh, Smo, and Gli1 protein in the synovial tissue of RA patients by immunohistochemistry were higher than health control (13). Shh is elevated in the plasma of RA patients with mild cognitive impairment (MCI), suggesting that it may be a biomarker for differentiating RA patients with MCI or without MCI (28). Furthermore, Shh signaling is activated both in the synovium of RA patients, in RA-FLS, and in rat RA synovial fibroblasts (RA-SF) (11).

In addition to the involvement of FLSs, damage of chondrocytes is also an important pathophysiological process in RA pathogenesis. In particular, cartilage damage is associated with irreversible disability in RA. Therefore, special attention should be given to the treatment of cartilage damage. The expression of Shh, Ptch1, Smo, and Gli1 proteins increased in AIA rats cartilage tissue, and the level of these proteins were proportional to the degree of chondrocyte damage (14). Moreover, angiogenesis is the fundamental cause of persistent synovial and chronic damage in RA. Apoptosis inhibition of microvascular endothelial cells may accelerate proliferation of synovial micro vessels. In the endothelial cells of RA synovial tissues, Smo expression was significantly elevated. The Shh pathway regulates apoptosis of endothelial cells through Smo protein (15).

There is also cross-linking between the Hh signaling and other pathways. Smo is associated with the proliferation of RA-FLSs and participates in the migration of RA-FLSs by activating the Rho GTPase signaling pathway (29). Shh signaling mediates proliferation and migration of RA-FLSs via the mitogen-activated protein kinases/extracellular signal-regulated kinases (MAPK/ERK) signaling (30). Besides, there is an interaction between c-Jun N-terminal kinase (JNK) signal and Shh signal in RA-FLS. Shh activation promotes the phosphorylation of JNK and c-jun, on the contrary, blocking Shh inhibits the activation of JNK signal. In the presence of JNK inhibitor, c-jun phosphorylation stimulated by Shh agonists was inhibited, indicating that Shh promoted the proliferation, migration and invasion of FLSs in a JNK-dependent manner (31).

### Osteoarthritis

Osteoarthritis (OA) is a degenerative disease characterized by pain and loss of joint function and is the most common cause of chronic disability in adults. Therefore, early diagnosis and effective treatment are crucial for OA. There is currently no effective treatment for OA. Therefore, a better understanding of the pathogenesis of OA will be beneficial to improve our knowledge of the disease (32). The pathophysiological changes of OA are mainly manifested in the morphological, biochemical, molecular, and biomechanical changes of cells and extracellular matrix (ECM), resulting in the gradual destruction of articular cartilage, inflammation of the synovium, and changes in the periarticular bone, and finally the formation of bone hyperplasia and subchondral bone sclerosis (33). Hypertrophic chondrocyte differentiation and degradation of the ECM are closely associated with the development and progression of OA. Hypertrophic chondrocytes upregulate the expression of extracellular matrix degrading enzymes MMP-13 and ADAMTS-5 (34).

The Hh signaling is activated in OA and thought to influence chondrocyte differentiation as well as osteoblastogenesis (35). Olex A et al. identified Hh as the most strongly regulated and metabolic pathway in OA disease development through genetic data analysis (36). Furthermore, Smo missense variants may be a new loci associated with OA. It regulates the transduction of Hh signaling by affecting the binding of cholesterol to its extracellular structure, thereby increasing the risk of hip OA (37). Ihh signaling protein expression is elevated in both human and mouse OA and positively correlates with OA severity (16). At the same time, Ihh promotes the hypertrophic chondrocyte phenotype and modulates the expression of canonical markers type X collagen and MMP-13 (17). The pathological score of FJOA is positively related with Ihh-related genes and hedgehog interacting protein (HHIP) (18).

Temporomandibular joint (TMJ) OA is another type of OA that exhibits degeneration of the articular cartilage and bone of the mandibular condyle and glenoid fossa/protrusion. In TMJOA rats, Ihh signal-related protein levels in cartilage were elevated and showed a time-dependent relationship with the severity of cartilage degeneration, suggesting that activation of Ihh signaling is associated with cartilage damage (19). Ihh is also associated with osteophytes and matrix mineralization in TMJOA (20). Shimoyama, A’s results indicate that Ihh temporally and spatially regulates Runt-related transcription factor (RUNX)2, an essential transcription factor for osteoblastogenesis, through Gli2 and consequently stimulates osteoblast differentiation (21). Ihh signaling promotes collagen X transcription and expression of chondrocytes by binding Runx2, which subsequently calcifies chondrocytes and participates in the pathogenesis of OA (22). The Ihh–parathyroid hormone–related protein (PTHrP) axis maintains the stability of articular chondrocytes. PTH1R may have a protective effect on TMJOA cartilage after Ihh signaling inhibition (23). In addition, Shh signaling is also involved in cartilage damage in OA (24).

Interleukin-1β (IL-1β) is colocalized with MMP-13 to sites of cartilage degradation in OA joints and is a key mediator of OA pathogenesis (38). Inhibition of IL-1β may lead to enhanced expression of Shh and MMP-13 in OA (39). Interleukin 18 (IL-18) could be secreted by chondrocytes, which is a member of the IL-1 cytokine family (40). IL-1β stimulates IL-18 expression in chondrocytes (41). IL-18 exerts a pro-inflammatory effect on chondrocytes by affecting their response to matrix degrading enzymes and matrix components, with the Hh pathway being involved in this process (42). However, other *in vitro* studies have shown that Ihh does not cause degradation of ECM in healthy chondrocytes even in the presence of IL-1β and that IL-1β downregulated the expression of Ihh. Thus, there may be other factors other than IL-1β involved in the degradation of OA ECM by Hh. Primary cilia are involved in the development and maintenance of articular cartilage and regulated Hh signaling (43). Depletion of primary cilia in articular chondrocytes activates Hh signaling, resulting in symptoms of early OA with a reduced ratio of Gli3 repressor to activator (44). Cholesterol is involved in OA chondrocyte development. Hh signaling is involved in cholesterol metabolism in human and mouse cartilage. The level of Gli expression was positively correlated with the intracellular cholesterol accumulation level and the severity of OA. Blocking cholesterol may reduce the severity of OA (45). Furthermore, activation of Smo is dependent on binding to sterols (46). Smo mutations in humans affect its binding to cholesterol and are associated with a high risk of hip OA (37).

### Systemic sclerosis

Systemic sclerosis (SSc) affects the skin and various internal organs including the lungs, the gastrointestinal tract and the heart. The accumulation of extracellular matrix (ECM) components in SSc is caused by increased production of ECM by activated fibroblasts (47). The profibrotic cytokine transforming growth factor-β (TGF-β) has been identified as a central mediator of fibroblast activation in SSc (48). The current study recognizes that TGF-β signaling and Wnt signaling are involved in the pathogenesis of SSc (49). Aberrant activation of these pathways in SSc potently stimulate fibroblast activation and collagen release, which results in tissue fibrosis. In addition, the Hh pathway is also activated in SSc. Hh is similar to TGF-β in that it induces myofibroblast differentiation and stimulates collagen release, which is also sufficient to induce fibrosis *in vivo (* 25). These findings confirm the involvement of Hh signaling in the aberrant activation of SSc fibroblasts. However, the relationship between these intracellular signals that stimulate ECM production is not fully understood. In skin biopsy tissues of SSc patients, the expression of Shh, Ptch1, Ptch2, Gli1, and Gli2 are all up-regulated, suggesting the activation of Hh pathway. The activation of it can stimulate collagen deposition and myofibroblast differentiation, and promote the development of fibrosis. Meanwhile, TGF-β and Wnt signaling promote the activation of this signaling through interaction in the animal model of SSc (25). Subsequent studies have shown that both Hh and TGF-β pathways intersect to Gli2, which promotes tissue fibrosis by integrating these signals. Moreover, they found that Gli2 is an important downstream agent of the profibrotic effects of TGF-β (50). HOTAIR regulates Gli2 expression in SSc myofibroblasts, and Gli2 also mediates the expression of profibrotic markers in Notch signaling (51). These findings may have translational implications as non-selective inhibitors of Gli2 are in clinical use and selective molecules are currently in development.

### Ankylosing spondylitis

Ankylosing spondylitis (AS) is characterized by inflammation and new bone formation (NBF) in the spine and joint (52). During the development of the vertebrate skeleton, chondrocytes are gradually replaced by bone. The Hh family is involved in ossification process (53), and Ihh is the main controller of endochondral ossification (54). Daoussis, D reported that the serum level of Ihh in AS patients was higher than RA patients and healthy controls, and its expression was reduced after TNF-antagonist treatment, suggesting that Ihh may be involved in the onset of AS and is important for the evaluation of AS prognosis. This finding may have both clinical and pathogenic implications (26). Relevant mechanistic studies have shown that Ihh is crucial for skeletal development and morphogenesis in vertebrates. It is mainly present in prehypertrophic chondrocytes and is involved in the proliferation and maturation of chondrocytes, but also in the maturation of osteoblasts in the inner cartilage layer (55). In addition, HLA-B27 is the most widely known genetic factor in AS, first reported in the early 1970s (56). HLA-B27 affects serum levels of key regulators of bone homeostasis. The serum Ihh level was significantly higher in HLA-B27 carriers than negative controls (57). Besides Ihh, after knockdown of Ptch1, the Hh pathway in chondrocytes is activated, which induces spinal fusion and spinal deformity. This is caused by massive proliferation of Ptch1 negative chondrocytes and impaired chondrocyte maturation thus leading to defects in endochondral ossification (53). In summary, the Hh pathway is participated in the development of AS disease, but there is a lack of studies on animal models of AS, and there are no clinical data from large samples to confirm the effectiveness of targeted inhibition of this pathway in the treatment of AS.

### Sjogren’s syndrome

Sjogren’s Syndrome (SS) is mainly involved in salivary glands and lacrimal glands. Several studies have demonstrated that Hh signaling participates in the morphogenesis of salivary glands and it has the potential to promote salivary glands (58, 59). Shh signaling is involved in the embryonic SMG branching morphogenesis. Cyclopamine decreased in branching and epithelial cell proliferation of SMG *in vitro (* 60). In the developing mouse submandibular gland, activation of Shh promotes cell polarization and lumen formation of submandibular gland (61). The expression of Shh in epithelium of mice embryonic salivary glands is induced by Edar/NF-κB pathway (62). Hh signaling is activated during functional regeneration in salivary glands after duct ligation of adult mice (63). Fiaschi M revealed that Gli1 has the ability to regulate salivary gland differentiation and promote ductal epithelial cell proliferation (64). Finally, the investigators also confirmed that the expression patterns of Shh pathway are highly similar during SG development in humans and mice, suggesting that the molecular mechanism regulating SG morphogenesis may be conserved (65). Therefore, targeting inhibition of Hh signaling may be a new direction for the treatment of SS.

## Treatment of rheumatic diseases by targeting the Hh signaling pathway

### Rheumatoid arthritis

With the intensive study of the Hh signaling in RA, the targeted therapy is also gradually increasing. After the administration of cyclopamine, a specific inhibitor of Shh pathway, the proliferation and expression of Shh, Smo, Gli1 mRNA were both reduced in RA-FLSs (13). Gli-antagonist 61 (GANT61) is a Gli specific inhibitor, it inhibited proliferation in a dose-dependent manner and the apoptosis rate of RA-FLSs was increased as well (11). Likewise, GANT61 reduced the level of Shh protein and inhibited proliferation of FLSs in CIA rats (67, 68). GDC-0449 is a Smo antagonist, using it or Smo siRNA can suppress the proliferation of FLSs, and the cell cycle-related proteins changed (69). Indicating that Shh pathway regulates FLSs in a Smo-dependent manner, and Smo may be a new target for RA therapy in the future. Ptch1 regulates Hh signaling negatively and the methylation level of it can affect the proliferation of FLSs. Methyl-CpG-binding protein 2 (MeCP2) overexpression results in an increase in overall methylation levels (90). The Ptch1 gene methylation level was increased in FLSs of adjuvant arthritis (AA) rats, which was related to the activation and inflammation of FLSs, and its methylation level was down-regulated after the administration of DNA methylation inhibitor. Knockdown of MeCP2 using siRNA added Ptch1 expression in AA FLSs. Increased expression of Ptch1 inhibited the activation of Hh pathway and decreased the expression of Gli1 and Shh (70). 5-(4-Chlorophenethyl) imino-7-O-acetyl-4-chlorophenethylamine hesperetin, a species of dihydroflavone, attenuates AA-FLSs inflammation by reducing the methylation level of the Ptch1 gene (71). Given that hypermethylation of the Ptch1 gene is associated with sustained FLSs activation and inflammation in AA rats, reducing the methylation level of it can not only regulate the FLSs inflammatory response but also inhibit the excessive proliferation of FLSs. Tumor necrosis factor (TNF)-like ligand 1A (TL1A) is a member of the TNF superfamily (TNFSF) ligands, which could have an influence on RA-FLSs through binding to TNFR2. TL1A significantly increased the expression of Ihh and its receptor Ptch1, 2 in RA-FLSs. However, TNFR2 antagonists can reduce the above genes notably. The enhanced migratory property of FLSs after stimulating with TL1A suggests that TL1A regulates the migration and Ihh signaling of RA-FLSs by TNFR2 (72). These findings may introduce a potential therapeutic strategy for targeting Ihh/Ptch1, 2 axes and control RA-FLSs pathological synovial invasion and suppress their undesired action. Furthermore, they confirmed the importance of clinical usage of anti-TNF drugs in patients with RA.

As for chondrocytes, Cyclopamine effectively decreased the expression of Shh, Ptch1, Smo, and Gli1 genes in AIA rats (14), while reducing joint inflammation and cartilage damage, decreasing the levels of pro-inflammatory factors in serum, and increasing the levels of COII in articular cartilage. Furthermore, cyclopamine inhibits apoptosis of chondrocytes, which may be related to its mechanism of alleviating cartilage damage (66). Transient receptor potential melastatin-like seven channel (TRPM7) has been reported to be associated with apoptosis. Its expression is elevated in chondrocytes and articular cartilage of AA rats, and inhibition of it can attenuate chondrocyte apoptosis and articular cartilage damage by modulating Ihh signaling (73).

In the endothelial cells of RA synovial tissues, Cyclopamine significantly reduced the expression of Shh pathway-related proteins in EA.hy926 endothelial cells, decreased cell viability and survival, and promoted apoptosis. Similarly, Smo-siRNA inhibited the proliferation of endothelial cells and promoted its apoptosis (15). Scube proteins are associated with endothelial cell inflammation and angiogenesis. More importantly, Scube promotes the activation of Hh signaling, and these features suggest that Scubes may be involved in inflammatory and angiogenesis of RA, and may be an ideal target for anti-angiogenic therapy in RA (91). Hypoxia-inducible factor 1 (HIF-1) α is also associated with RA synovitis and angiogenesis (92). AMSP-30m, a novel HIF-1 alpha inhibitor, inhibits angiogenesis in many tumor cells (93). In the AIA model, it had strong anti-arthritic and anti-inflammatory effects, inhibited SFs proliferation and promoted its apoptosis. AMSP-30m exerted its anti-angiogenic effect by reducing the expression of synovial vascular endothelial growth factor (VEGF) and the number of blood vessels. In addition, it inhibited the activation of Shh pathway (74). Collectively, the HIF-1α inhibitor AMSP-30m may exert an effective anti-arthritic effect by promoting synovial apoptosis, reducing angiogenesis and inhibiting the Shh pathway. Annexin a2 (Axna2) is an important mediator of pannus formation in RA. The expression of Axna2 and Axna2 receptor (Axna2R) is elevated in RA patients. In CIA model, overexpression of Axna2 promotes the development of arthritis, especially the formation of pannus. Meanwhile, Axna2 activates and amplifies the expression of Hh pathway and its downstream VEGF, angiopoietin-2 (Ang-2), and matrix metalloproteinase-2 (MMP-2) by binding to Axna2R, promoting the proliferation of HUVEC and ultimately leading to the formation of pannus (94). Therefore, inhibition of Axna2 may be a new potential measure for the treatment of RA.

### Osteoarthritis

Given the important role of the Hh signaling in the development of OA, researchers have explored its use as a therapeutic target. Zhou, J’s study demonstrates that knockdown of Ihh prevents OA progression by inhibiting chondrocyte hypertrophy and collagen type X, MMP-13 and Runx2 expression (75). The study also found that treatment with an Ihh inhibitor or Smo-siRNA could inhibit Hh signaling and reduce the severity of OA (16), consistenting with the results of Wei, F et al. (17). Activation of Notch signaling inhibits Hh signaling in OA. Studies have found that when Notch signaling is inhibited, Hh signaling is activated, thereby promoting chondrocyte hypertrophy and bone hyperplasia (95). Selective inhibition of Hh signaling in Gli1^+^ osteoblastic progenitor cells reduces subchondral local immune inflammatory responses and attenuates articular cartilage degeneration in TMJOA mouse model, providing a potential method for TMJOA treatment (76).

There are already Hh inhibitors used in the treatment of tumors, which provides some basis for the use of Hh inhibitors in OA. LDE223 is a Smo-specific small molecule inhibitor that inhibits chondrocyte differentiation, reduces the expression of type X collagen, and inhibits bone hyperplasia in mice (77). Palmatine (Pal), a member of the protoberberine class of isoquinoline alkaloids, is a structural analog of berberine. It has a protective effect on cartilage and may inhibit MMPs by inhibiting the Hh signaling (78). *Phlomis umbrosa* extract (PUE) has been used in the treatment of RA (79). PUE also can improve joint pathology in animal models of OA by modulating the Shh signaling pathway (80). Ipriflavone also can attenuate the degeneration of cartilage by blocking the Ihh pathway (81). Prochondrocytes express the kappa opioid receptor (KOR), which prevents the onset of cartilage degeneration (96). It was found that the selective KOR agonist JT09 reduced cartilage loss and prevented degenerative changes in joints, while reducing the expression of Hh signaling components in cartilage (82). Combined treatment of GANT-61 and indomethacin reduced cartilage damage and decreased levels of TNF-α, IL-2, and IL-6 in OA (83). The ectonucleotide pyrophosphatase/phosphodiesterase 1 (Enpp1) is an inhibitor of pathological calcification that inhibits ectopic joint calcification and maintains articular chondrocytes by inhibiting Hh signaling, whereas Enpp1 expression is diminished in articular cartilage of human and mouse OA (84). miR-1 is lowly expressed in human OA joint tissues, and overexpression of it inhibits the development of OA by inhibiting Ihh signaling (85). The activation of Hh signaling plays an important role in the occurrence and development of OA, and a large number of studies have proved that targeting and blocking this pathway can treat OA (97, 98), laying a certain foundation for its future clinical translation.

### Systemic sclerosis

As a driver of fibroblast activation, chloride intracellular channel 4 (CLIC4) expression is elevated in fibroblasts from SSc patients. It is associated with increased activation of TGF-β (99). Investigations revealed the expression of CLIC4 in normal dermal fibroblasts was driven by TGF-β/SMAD3 together with Wnt3a/β-catenin and Smo/Gli signaling, and inhibition of Gli reduced CLIC4 expression (86). The hedgehog acyltransferase (HHAT) catalyses the attachment of palmitate onto Shh (100). Knockdown of HHAT inhibits TGF-β-induced Hh signaling expression, while suppressing fibroblast activation and tissue fibrosis (87). Inhibition of Hh signaling by targeting HHAT may become a new approach for SSc antifibrotic therapy. LDE223 is a selective small-molecule inhibitor of Smo, which can lighten bleomycin-induced dermal fibrosis and inhibit the aberrant activation of Hh pathway (88). In lung tissue of SSc-ILD patients, Hh pathway is activated, pirfenidone intervention reduces its expression (89). In all, the Hh signaling contributes to the aberrant activation of SSc fibroblasts, and antifibrotic therapy targeting it is expected to improve the prognosis of SSc patients, however, there is still a long way to go for true clinical translation.

It is not difficult to see that there have been many studies on the targeting treatment of the pathway. However, it is worth noting that these studies are all animal or *in vitro* experiments, and there is no relevant clinical research. Moreover, research on specific inhibitors of this pathway is still missing. Expression of target genes can be inhibited by si-RNA, thereby inhibiting the activation of this pathway, but this method is difficult to apply to patients. GANT61, GDC-0449 and LDE223 are specific small molecule inhibitors of this pathway, at the moment there is little/no knowledge on the pharmacokinetics (e.g. solubility, metabolism, etc.) of these agents and its toxicity. Cyclopamine is the most studied inhibitor of the Hh pathway, and its clinical use is limited due to its insolubility in water and organic solvents. The inhibitory effect of the above drugs on this pathway is not specific, and there is a lack of research on its adverse reactions in rheumatism.

## Conclusion

**Figure 2:**
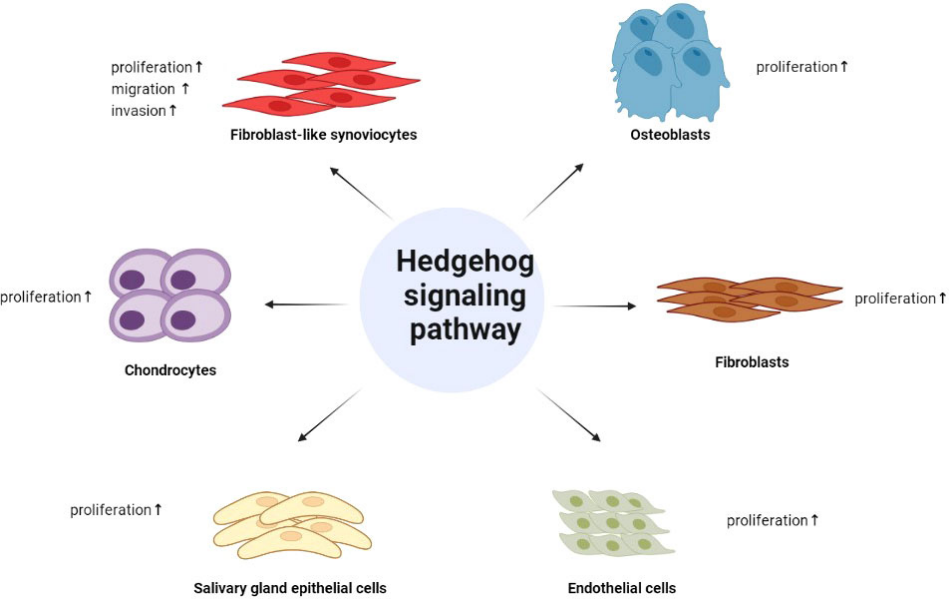
The role of Hedgehog signaling in rheumatic diseases. Hedgehog signaling pathway is involved in the inflammatory proliferation of FLSs, the differentiation and proliferation of chondrocytes, osteoblasts, and fibroblasts, and can stimulate the proliferation of endothelial cells and salivary gland epithelial cells.

With the deepening of the current research on the Hh signaling, its role in rheumatic diseases has also been verified. In conclusion, the aberrant activation of it is involved in the inflammatory proliferation of FLSs, the differentiation and proliferation of chondrocytes, osteoblasts, and fibroblasts, and can stimulate the proliferation of endothelial cells and salivary gland epithelial cells, providing a new perspective for the treatment of multiple rheumatic diseases (**Figure 2**). What is even more exciting is that some inhibitors of the Hh pathway have been applied in the clinical treatment of certain tumors, and many drugs have entered the clinical research of various tumor treatments, so the application of these drugs in rheumatic diseases will also be not far. At the same time, it is even more necessary for us to conduct in-depth research on Hh signaling pathway to provide new ways for the treatment of rheumatic diseases.

## Author contributions

This article is mainly written by YS. HX wrote part of the manuscript and proofread the manuscript. JK and LB helped us collect literature information and draw pictures. LZ reviewed the manuscript and proposed final revisions. All authors contributed to the article and approved the submitted version.

## Funding

This work was supported by the National Natural Science Foundation of China [grant number 81771768] and by the basic research project of Shanxi Science and Technology Department [grant number 202103021224342].

## Conflict of interest

The authors declare that the research was conducted in the absence of any commercial or financial relationships that could be construed as a potential conflict of interest.

## Publisher’s note

All claims expressed in this article are solely those of the authors and do not necessarily represent those of their affiliated organizations, or those of the publisher, the editors and the reviewers. Any product that may be evaluated in this article, or claim that may be made by its manufacturer, is not guaranteed or endorsed by the publisher.
